# The Surgical Diseases of Children

**Published:** 1898-06

**Authors:** 


**The Surgical Diseases of Children.** By EDMUND OWEN, M.B. Third Edition. Pp. xiv., 504. London: Cassell and Company, Limited. 1897.

In reviewing a book on diseases of children it is necessary to clear the way, by stating that we consider that such books—as usually written—are unnecessary. Many of the diseases which are common in childhood, and even those which are peculiar to that age, such as rickets, are often much better described in a work on general medicine or surgery. If the writer of a book on diseases of children would confine himself to the peculiarities of disease as manifested in childhood, his work would be of real value. He might take as a model Charcot's work on disease as it is modified by old age. But when we take up a book on diseases of children, we find as a rule a text-book of all diseases met with at that period of life. The value of such a work will depend on the skill of the writer in describing the diseases, and his experience in treating them; and such experience will only be gained by one who has been on the staff of a large children's hospital. The work will be of value mainly from the experience of the writer in this special line of practice. In this way the book before us is of particular value, for Mr. Owen is well known as an able surgeon, with a very large experience of surgical diseases of children, and hence the opinions he expresses are well worth careful attention. But the work as a text-book of surgery—as indeed it is—should in no way replace the well-accepted text-books of the present day, even in the study of diseases which occur in children: for instance, the subject of talipes is far better described in one of our large text-books of surgery, such as Erichsen or Treves, than it is in Mr. Owen's book, and yet it is essentially a deformity demanding treatment in childhood. In Mr. Owen's description of the deformity, there is no mention whatever of the condition of the structures in the deformed foot. A work on surgical diseases of children should supplement, and not replace, a study of such diseases in the larger works on general surgery, and should supplement them only in so far as it sets forth the peculiarities of disease occurring in childhood.

As a text-book of surgical diseases of children, Mr. Owen's work is a successful attempt to give in a small space a general outline of such diseases: it is by no means an exhaustive treatise. It would be better not to write on a subject at all than to treat it so inadequately as the important subject of thrombosis of the lateral sinus is dealt with. But here the author includes a disease by no means confined to children, and it is a good illustration of the mistake we pointed out in our remarks on what the scope of such a work ought to be. Then, again, in a book devoted to the diseases of children, surely the treatment of congenital dislocation of the hip-joint, by operation or otherwise, might have received full consideration, as it has filled so many pages of our journals with able articles for years past, but we are disappointed to find that this is not so.

This book is clearly written and remarkably well illustrated, and even includes so modern a picture as a skiagram of a case of tuberculous hip-disease. The author has evidently revised and added to the work in the light of recent discovery and increase of knowledge. There is also a vast improvement in the general appearance of the work. This edition has larger type and a much better cover than the previous issues had.

